# Design of a Novel Artificial Atlanto‐Odontoid Joint and Its Anatomical and Radiological Studies Following Transoral Pharyngeal Approach Arthroplasty

**DOI:** 10.1111/os.70088

**Published:** 2025-07-22

**Authors:** Shengguang Lei, Lingyong Jing, Xijiong Chen, Zhiwei Gan, Jianbin Zhong, Liming Yu, Yong Hu

**Affiliations:** ^1^ Health Science Center Ningbo University Ningbo China; ^2^ Department of Orthopedic Trauma Zhedong Orthopedic Hospital Ningbo China; ^3^ Department of Spine Surgery Ningbo No. 6 Hospital Ningbo China; ^4^ Department of Spine Surgery Ningbo Ninth Hospital Ningbo China

**Keywords:** arthroplasty, artificial atlanto‐odontoid joint, atlantoaxial instability, transoral pharyngeal approach, upper cervical spine

## Abstract

**Objective:**

For atlantoaxial joint disorders, traditional surgical approaches often presented challenges such as significant trauma and prolonged recovery. Therefore, it was crucial to explore safer and more effective surgical alternatives. The primary aim of this study was to investigate the anatomical safety and feasibility of artificial atlanto‐odontoid joint (AAOJ) replacement via a transoral pharyngeal approach, through simulated surgical procedures and postoperative anatomical and radiological studies.

**Methods:**

The novel AAOJ replacement surgery was simulated on 18 fresh adult cadaveric head and neck specimens, and relevant anatomical parameters were measured. Postoperatively, the specimens underwent X‐ray and CT scans, and software was used to measure the relevant parameters of the fixation screws. The spatial relationships between the atlantoaxial components, fixation screws, and critical anatomical structures were also examined. The comparison of parameters between the left and right sides was conducted using paired‐sample *t*‐tests.

**Results:**

The transoral pharyngeal approach provided adequate exposure, clear surgical visualization, and sufficient working space. Anatomical measurements showed that the width of the anterior arch bone window of the atlas was (13.8 ± 0.7) mm; the width of the vertebral body bone window of the axis was (11.0 ± 0.4) mm; the distance between the insertion points for the atlas screws was (28.2 ± 4.0) mm; the distance from the atlas insertion points to the lateral joint edge of the atlanto‐axial joint was (5.2 ± 0.9) mm; the distance between the insertion points for the axis screws was (16.8 ± 1.6) mm; and the distance from the axis insertion points to the lateral joint edge of the atlanto‐axial joint was (7.7 ± 0.9) mm. Radiological measurements showed that the screw trajectory length of the lateral mass screw in the atlas was (21.5 ± 2.8) mm, the outward insertion angle was (13.2 ± 2.5)°, and the caudal insertion angle was (3.5 ± 1.1)°; for the pedicle screw of the axis, the screw trajectory length was (29.8 ± 2.8) mm, the outward insertion angle was (20.7 ± 2.8)°, and the caudal insertion angle was (16.6 ± 2.7)°. The prosthesis was precisely fitted to the upper cervical spine, with adequate safety distances between the atlantoaxial components, fixation screws, and critical anatomical structures such as the foramen transversarium, vertebral artery groove, and spinal canal.

**Conclusions:**

The transoral pharyngeal approach for novel AAOJ replacement is anatomically safe and feasible.

## Graphical abstract

The implantation of the novel artificial atlanto‐odontoid joint (AAOJ) is both safe and convenient, and it preserves the normal range of rotational, flexion‐extension, and lateral bending motion between the atlas and axis.

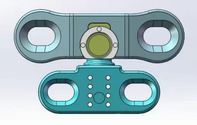

## 1 Introduction

The upper cervical spine of the human body consists of the atlas (C1) and axis (C2), which together form the atlantoaxial joint. This joint includes the lateral atlantoaxial joints (plane joints) on either side, as well as the central atlantoodontoid joint (pivot joint). The coordinated function of these structures makes the atlantoaxial joint one of the most mobile joints in the human spine [1]. However, the high degree of mobility also predisposes the joint to instability. Atlantoaxial instability and dislocation are common conditions in spinal surgery, often resulting from congenital malformations, high‐energy trauma, or conditions such as upper cervical spine tumors, tuberculosis, and rheumatoid arthritis [2, 3].

Decompression, bone grafting, and internal fixation have been the most widely used surgical techniques for treating atlantoaxial instability and dislocation since the last century. However, long‐term follow‐up results of this procedure indicate that patients may experience varying degrees of loss of cervical motion, particularly in rotational function [4, 5]. Additionally, adjacent segment degeneration of the cervical spine can occur, leading to a decline in postoperative quality of life [6]. In contrast, decompression alone often exacerbates instability between the atlas and axis, with suboptimal results in mid‐ to long‐term follow‐ups [7]. In the 21st century, with the emergence of the non‐fusion concept for the upper cervical spine and continuous advancements in surgical techniques, there have been significant developments in the surgical management of atlantoaxial instability. Among these, the artificial atlanto‐odontoid joint (AAOJ) replacement has become an important treatment option. It not only restores the stability of the atlantoaxial joint but also successfully preserves its normal range of motion while maintaining joint stability.

In 2003, Cao et al. [8] introduced an AAOJ design consisting of two components: an artificial anterior arch of the atlas and an artificial odontoid process. This design allowed for near‐normal motion of the atlantoaxial joint postoperatively [9, 10]. However, subsequent biomechanical testing revealed that the stiffness of the prosthesis differed significantly from that of the normal atlantoaxial joint, indicating suboptimal biomimetic performance. In 2004, Tan et al. [11] proposed a method involving the resection of the transverse ligament and the use of a steel plate with a semi‐circular joint surface to reconstruct the stability of the atlantoodontoid joint. This design theoretically better preserved the motion and stability of the atlantoaxial joint without further damaging other ligamentous structures. However, the in vivo experimental results were less than satisfactory, and the procedure had a limited scope of indication, requiring the odontoid process to remain intact [12]. In 2008, Lu et al. [13, 14] further modified this design by adding a limiting structure to precisely control the range of motion, but it still failed to meet expectations in clinical practice.

Since 2006, our research group has been dedicated to the design and development of the AAOJ. Our design approach also follows a two‐component concept, simulating the anterior arch of the atlas and the odontoid process, mimicking the unique “pivot joint” structure of the normal atlantoodontoid joint. Additionally, the base of the axis component is designed with a porous, hollow structure, which allows for bone grafting during surgery to promote bony fusion and enhance prosthesis stability. This innovative feature differentiates our design from other alternatives [15]. However, this AAOJ allows only for rotational motion and does not facilitate flexion, extension, or lateral bending, leading to the loss of some normal functional movements of the atlantoaxial joint [16]. Therefore, developing a safe, convenient, and highly biomimetic AAOJ remains a significant challenge.

For nearly two decades, our research group has been committed to the development and refinement of the AAOJ. Through continuous improvement, we have developed a new generation of AAOJ with the aim of achieving better surgical outcomes. We have optimized the original “pivot joint” design into a “ball‐and‐socket joint” structure and incorporated limiting structures both within the socket and above the ball. This modification not only preserves the rotational motion of the atlantoodontoid joint but also retains its flexion, extension, and lateral bending functions. In this study, we implanted the newly designed AAOJ via a transoral approach into the upper cervical spine of adult cadaver specimens, followed by anatomical and imaging analysis of the prosthesis and atlantoaxial joint postoperatively. The objectives of this study are as follows: (i) to evaluate the feasibility of implanting the novel AAOJ through a simulated surgical procedure; (ii) to assess the anatomical safety of the novel AAOJ implantation based on the anatomical and radiological parameters measured, and to establish safety thresholds.

## 2 Methods

### 2.1 Design of the Novel AAOJ

**FIGURE 1:**
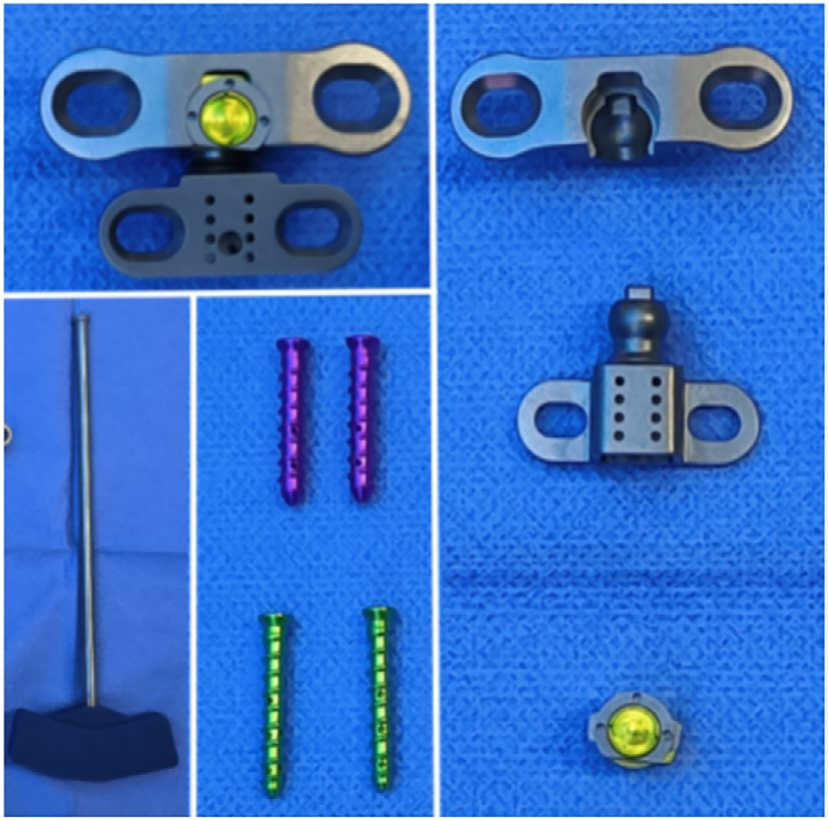
Photograph of the novel AAOJ and its components.

In order to preserve the maximum functional mobility of the atlantoaxial joint after AAOJ implantation, we have modified the original design of the AAOJ, resulting in a novel version (Patent No: ZL 2024 20756611.8). This new AAOJ consists of three main components: the atlantal component, the axial component, and the locking cap (Figure 1).

The atlantal component is composed of a biomimetic “ball‐and‐socket” structure that imitates the transverse ligament and lateral wings designed to replicate the anterior arch of the atlas. The central “ball‐and‐socket” joint has a maximum depth of 13 mm, while the two lateral elliptical‐shaped fixation plates are inclined at a posterior angle of 12°, ensuring a tight fit with the anterior arch of the atlas. The highest point of the lateral edges reaches 12 mm, with a width of 37.85 mm and a plate thickness of 2 mm. Each lateral side of the component contains one elongated elliptical‐shaped fixation hole designed to accommodate different screw entry points according to individual anatomical variations. Each fixation hole can be secured using a hollow lateral hole screw.

The axial component comprises a base, two lateral fixation plates, and a biomimetic serrated protruding ball. The lateral fixation plates are 2 mm thick and elliptical in shape, with the highest point on the outer edge reaching 9.5 mm and a width of 27 mm. The base is designed with an isosceles trapezoidal shape, with equal width on both sides and a hollow, mesh‐like, wedge‐shaped structure that has a 5.5° anterior‐to‐posterior slope. The base dimensions are 10 mm in width and 9.5 mm in thickness. This wedge shape facilitates a 5° posterior angulation of the atlantal component when assembled, closely mimicking the natural angulation between the atlas and axis. Additionally, each lateral fixation plate of the axial component includes an elongated elliptical screw hole for fixation.

The locking cap is designed with a sliding rail structure between it and the atlantal component. By using a custom handle, the locking cap's protruding section can be slid into the pre‐formed rail of the atlantal component to achieve a secure locking mechanism.

All components of the novel AAOJ are fabricated from titanium alloy, manufactured by Shandong Weigao Orthopedic Medical Devices Co. Ltd.

### 2.2 Simulated Surgery

Eighteen fresh adult cadaveric head and neck specimens (preserved from the head to the C7 vertebra) were selected for the study, provided by the Anatomy Laboratory of the Health Science Center, Ningbo University. Among these, 11 were male specimens and 7 were female specimens, with ages ranging from 36 to 63 years (mean age: 51 years). Specimens were carefully examined for any congenital malformations, fractures, tumors, or other pathological conditions using both visual inspection and X‐ray imaging. Following the examination, the specimens were immediately subjected to replacement surgery. The surgery was performed by one experienced spinal surgeon, assisted by two surgical assistants.

**FIGURE 2:**
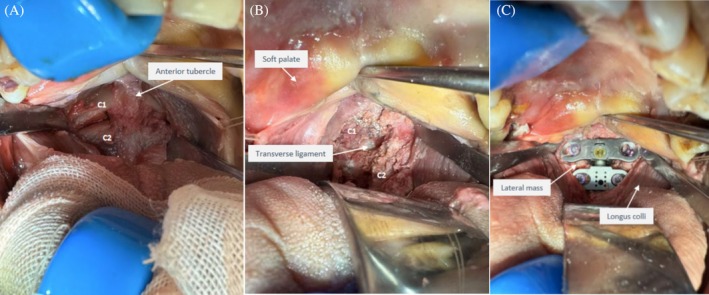
Replacement of the novel artificial atlanto‐odontoid joint (AAOJ) (A) Surgical exposure area; (B) Creation of the atlanto‐axial bone window; (C) Completion of the novel AAOJ implantation.

The experimental simulated replacement surgery was performed following a series of steps as outlined below: (1) *Position, Incision, and Surgical Exposure*: The patient was placed in a supine position with the head and neck slightly extended and fixed using a head collar. A transoral approach via the anterior oropharyngeal route was employed. The surgical field was disinfected and draped in accordance with standard procedures, with the oropharyngeal region exposed. A fine urinary catheter was inserted through the nasal cavity and sutured to the uvula. The catheter was then gently pulled outward, causing the soft palate and uvula to be displaced into the nasopharyngeal space, thus clearing the surgical area. A DAVIS retractor was used to open the oral cavity, providing clear and complete visualization of the posterior pharyngeal wall. The finger was used to palpate the midline bony prominence, which was identified as the anterior tubercle of the atlas. A vertical incision, approximately 4.5 cm in length, was made along the anterior tubercle of the atlas, extending from the inferior edge of the slope to the inferior margin of the axis. The incision was carried out through the buccopharyngeal fascia, prevertebral fascia, prevertebral muscles, anterior longitudinal ligament, longus capitis muscle, and longus colli muscle, sequentially, until the anterior tubercle of the atlas, anterior arch of the atlas, anterior surface of the lateral masses of the atlas, axis vertebral body, and anterior surface of the axis pedicle were fully exposed (Figure 2A). (2) *Creation of the Atlantoaxial Bony Window*: Using high‐speed diamond burrs, fine‐toothed bone forceps, lamina bone forceps, and nucleotomy forceps, the anterior arch of the atlas, odontoid process, and cruciate ligament were progressively removed until the spinal canal was clearly exposed. The inner surface of the lateral masses of the atlas was smoothed to create a gap of approximately 13–15 mm between the left and right sides of the anterior arch of the atlas, ensuring that the atlanto‐occipital joint and the bilateral atlantoaxial lateral joints above were not damaged. Subsequently, using a high‐speed diamond burr and bone forceps, a rectangular bone groove was created in the upper‐middle portion of the axis vertebral body. A spherical diamond burr was used to smooth the bone surfaces of the groove until the dimensions of the groove were suitable for the insertion of the axis components, measuring 9.5 mm in height, 10 mm in width, and 9.5 mm in depth (Figure 2B). (3) *Implantation of the Prosthesis*: Bone fragments generated during the groove creation in the axis were collected and implanted into the base of the axis component. First, the atlas component was placed into the pre‐formed bone groove in the anterior arch of the atlas. The fixation holes on both sides were prepared by opening, tapping, and probing the screw pathways. Then, one 4.0 mm diameter hollow lateral hole screw was used on each side for temporary fixation (entry point: central point of the anterior surface of the lateral mass of the atlas; screw direction: along the long axis of the lateral mass of the atlas, inclined posterolaterally). The axis component was then placed, with the spherical part inserted horizontally into the ball‐and‐socket joint of the atlas component and the base inserted into the preformed groove on the anterior surface of the axis vertebral body. The fixation plate of the axis component was secured temporarily on both sides with a 3.5 mm diameter hollow lateral hole screw inserted into the pedicle of the axis vertebra (entry point: central point of the anterior surface of the axis pedicle; screw direction: along the long axis of the axis pedicle, inclined posterolaterally). Finally, a locking cap was placed on top of the spherical part to secure it, and the corresponding screws were tightened (Figure 2C). The surgical incisions were then closed in layers.

### 2.3 Anatomical Measurements of the Specimens

**FIGURE 3:**
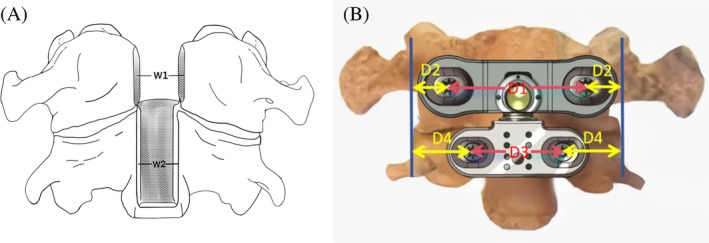
Schematic diagram of anatomical measurement parameters (A) W1 represents the width of the anterior arch bone window of the atlas; W2 represents the width of the vertebral body bone window of the axis; (B) D1 represents the distance between the insertion points for the atlas screws; D2 represents the distance from the atlas insertion points to the lateral joint edge of the atlanto‐axial joint; D3 represents the distance between the insertion points for the axis screws; D4 represents the distance from the axis insertion points to the lateral joint edge of the atlanto‐axial joint.

Intraoperative measurements were performed for the following parameters: (1) width of the anterior arch bone window of the atlas (W1); (2) width of the vertebral body bone window of the axis (W2, as shown in Figure 3A). Postoperatively, after complete removal of the cervical spine specimens and excision of surrounding soft tissues, the following parameters were measured using a digital caliper (precision 0.01 mm): (3) distance between the insertion points for the atlas screws (D1); (4) distance from the atlas insertion points to the lateral joint edge of the atlanto‐axial joint (D2); (5) distance between the insertion points for the axis screws (D3); (6) distance from the axis insertion points to the lateral joint edge of the atlanto‐axial joint (D4, as shown in Figure 3B). Each measurement was repeated three times per specimen, and the average value was recorded.

### 2.4 Radiological Observation and Measurement of the Specimens

After anatomical measurements, the cervical spine specimens underwent X‐ray examination (Definium 6000, GE, USA) and CT high‐resolution scanning (Optima CT670, GE, USA). The CT scanning parameters were set as follows: slice thickness 1 mm, slice interval 0.5 mm, pitch 0.938, tube voltage 120 kV, tube current 250 mA, window width 1000, and window level 300. The raw data obtained from the plain scan were transferred to the Sgi02 workstation for further processing. Two‐dimensional and three‐dimensional CT reconstructions were performed using multi‐plane reconstruction (MPR) and volume rendering (VR) techniques. The following parameters were measured on the reconstructed sagittal and axial images using 3D Slicer software: (1) the screw trajectory length of the lateral mass screw in the atlas (L1); (2) the insertion angulation of the lateral mass screw in the atlas in the outward direction (A1); (3) the insertion angulation of the lateral mass screw in the atlas in the caudal direction (A2); (4) the screw trajectory length of the pedicle screw in the axis (L2); (5) the insertion angulation of the pedicle screw in the axis in the outward direction (A3); (6) the insertion angulation of the pedicle screw in the axis in the caudal direction (A4). The anatomical relationship between the components of the novel AAOJ and critical structures such as the spinal canal, transverse foramen, and vertebral artery groove was observed to assess the accuracy and anatomical safety of screw placement.

### 2.5 Statistical Analysis

Data were analyzed using SPSS software (version 26.0; IBM, Armonk, NY, USA). Variables were expressed as mean ± standard deviation. A paired‐sample *t*‐test was performed to compare the parameters between the left and right sides. Statistical significance was set at *p* < 0.05.

## 3 Results

### 3.1 Simulated Surgery

The transoral pharyngeal approach provided sufficient exposure of the surgical site, with a clear operative field and adequate working space. The exposure range extended superiorly to the anterosuperior aspect of the foramen magnum and inferiorly to the lower margin of the axis vertebral body. The operative field could be further extended to C3 by enlarging the retractor opening, without causing dislocation of the temporomandibular joint. The process of establishing the atlantoaxial bone window was smooth, and, with careful manipulation and appropriate protection, no damage to critical structures such as the spinal cord and upper cervical sympathetic ganglia was observed. The bone debris generated during the groove formation was sufficient to fill the base of the axis component. Following exploration with a nerve dissector, the screw trajectories were confirmed to have intact four walls, without encroachment into the spinal canal or transverse foramen. Neither vertebral arch fractures nor screw breakage was observed during the approach and screw placement. After becoming familiar with the anatomy and procedural steps, the total surgical time was controlled within 1.84 ± 0.24 (1.5–2.3) hours.

### 3.2 Anatomical Measurements of the Specimens

The relevant anatomical data for the novel AAOJ implantation procedure, both intraoperatively and postoperatively, are presented in Table 1. No statistically significant differences were observed between the left and right side measurements for D2 (5.2 ± 0.9 and 5.2 ± 0.8 mm) and D4 (7.8 ± 0.8 and 7.7 ± 0.9 mm) (*p* > 0.05, *p* > 0.05). Therefore, the measurements from both sides were combined, and the mean values were calculated.

**TABLE 1:** Relevant anatomical parameters during and after the novel artificial atlanto‐odontoid joint implantation procedure (Mean ± SD, *n* = 18, mm).

Parameters	Results	Ranges
W1	13.8 ± 0.7	12.6–15.0
W2	11.0 ± 0.4	10.2–11.7
D1	28.2 ± 4.0	24.9–32.2
D2*	5.2 ± 0.9	3.6–6.8
D3	16.8 ± 1.6	14.2–19.2
D4*	7.7 ± 0.9	6.3–9.3

*Note*: *The bilateral measurements showed no statistically significant differences upon comparison (*p* > 0.05). W1 represents the width of the anterior arch bone window of the atlas; W2 represents the width of the vertebral body bone window of the axis; D1 represents the distance between the insertion points for the atlas screws; D2 represents the distance from the atlas insertion points to the lateral joint edge of the atlanto‐axial joint; D3 represents the distance between the insertion points for the axis screws; D4 represents the distance from the axis insertion points to the lateral joint edge of the atlanto‐axial joint.

### 3.3 Radiological Observation and Measurement of the Specimens

**FIGURE 4:**
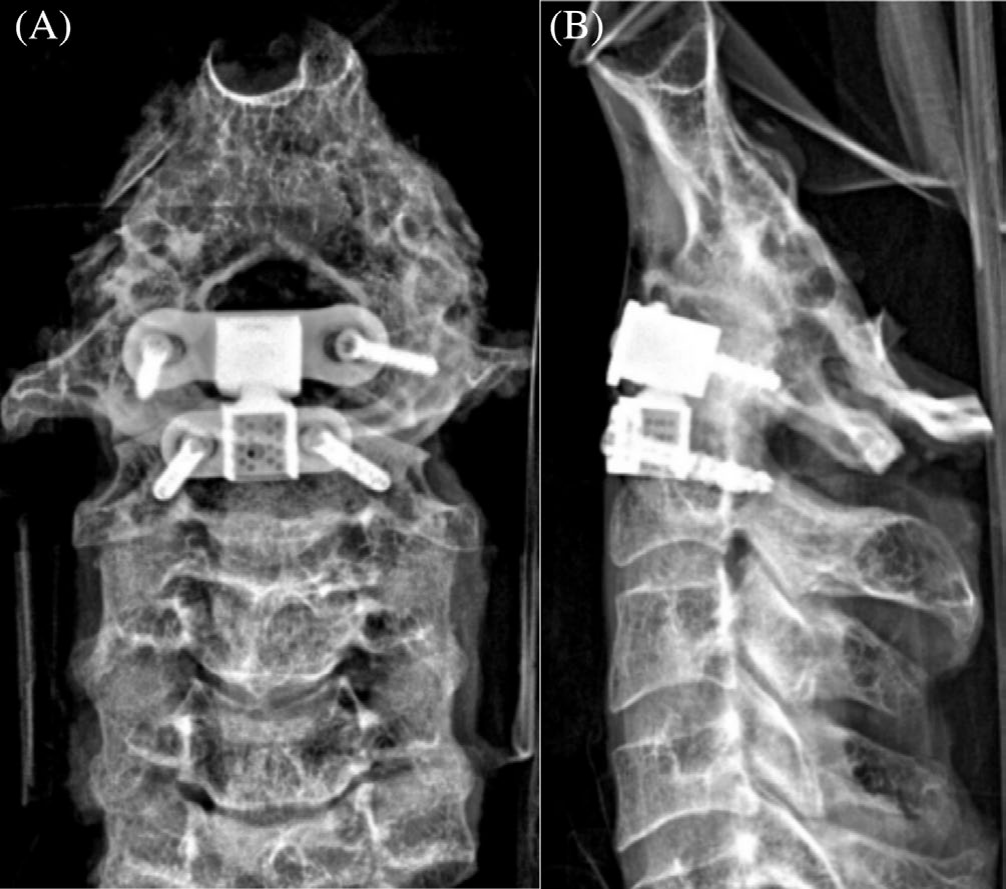
Postoperative X‐ray of the novel artificial atlanto‐odontoid joint (AAOJ) replacement (A) Anteroposterior view; (B) Lateral view.

**FIGURE 5:**
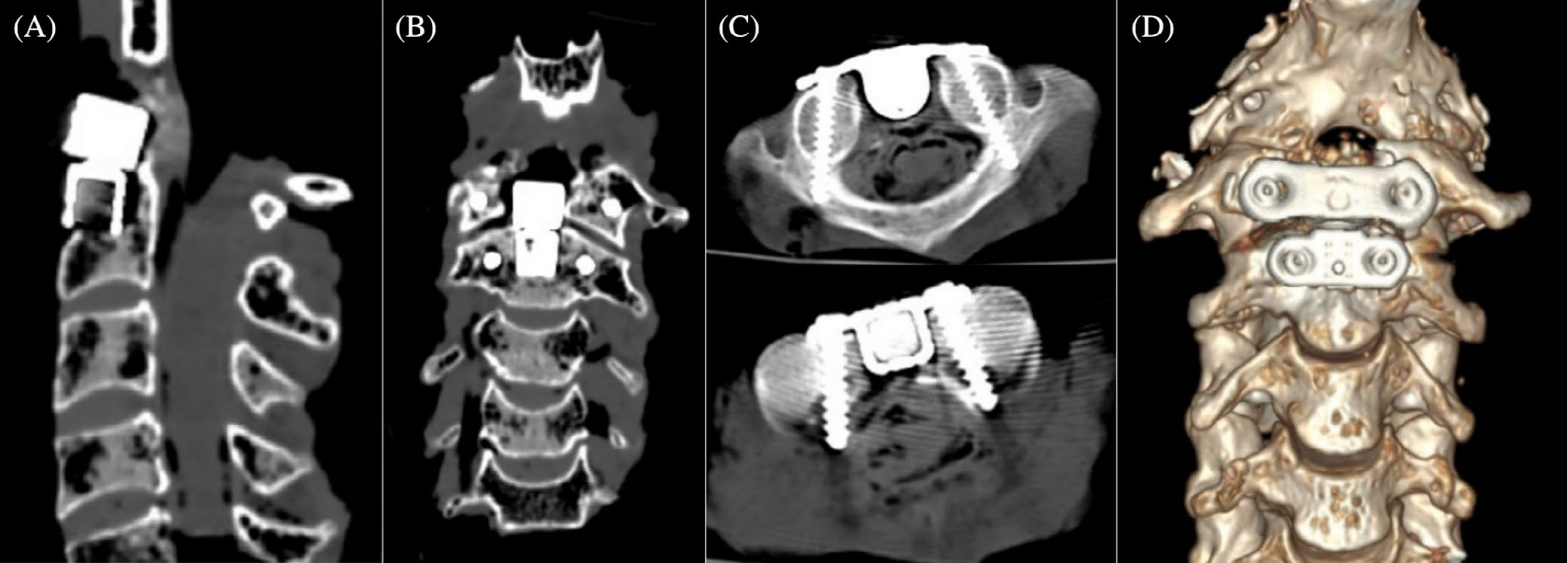
Postoperative CT scan and three‐dimensional reconstruction of the novel artificial atlanto‐odontoid joint (AAOJ) replacement (A) Sagittal view; (B) Coronal view; (C) Axial view of the atlas and axis; (D) Three‐dimensional reconstructed image.

Postoperative X‐ray and CT imaging results of the cervical spine specimens following the novel AAOJ implantation are shown in Figures 4 and 5, respectively. The measurements of the fixation screw trajectories for the atlantoaxial components are summarized in Table 2. Both X‐ray and CT imaging results demonstrated that the size and shape of the novel AAOJ atlantoaxial components were precisely adapted to the grooved atlantoaxial complex. The contact surface between the bone and the prosthesis was thoroughly congruent, exhibiting a high degree of tightness. The anterior margin of the atlantoaxial component aligned smoothly with the anterior margin of the lower cervical vertebral body, preserving the normal physiological lordosis of the cervical spine. The fixation screws of the atlantoaxial components were of appropriate length and correctly positioned within the target region, with the insertion angles consistent with the preoperative design. No screw loosening or displacement was observed, and no damage to surrounding structures was noted.

**TABLE 2:** Radiographic measurement parameters of screw trajectories following the novel artificial atlanto‐odontoid joint implantation procedure (Mean ± SD, *n* = 18).

Parameters	Left side	Right side	*p*	Combined
L1 (mm)	21.8 ± 1.7 (18.8–24.4)	22.2 ± 1.6 (19.0–24.2)	0.088	21.5 ± 2.8 (18.8–24.4)
A1 (°)	13.0 ± 2.2 (9.5–16.6)	13.4 ± 2.9 (9.3–16.7)	0.533	13.2 ± 2.5 (9.3–16.7)
A2 (°)	3.4 ± 1.2 (1.4–5.2)	3.5 ± 1.1 (1.6–5.3)	0.250	3.5 ± 1.1 (1.4–5.3)
L2 (mm)	29.9 ± 2.9 (24.8–34.2)	29.7 ± 2.7 (25.0–33.9)	0.104	29.8 ± 2.8 (24.8–34.2)
A3 (°)	20.6 ± 2.8 (15.0–25.0)	20.7 ± 2.9 (14.9–25.2)	0.408	20.7 ± 2.8 (14.9–25.2)
A4 (°)	16.6 ± 2.8 (10.5–21.0)	16.7 ± 2.6 (10.8–20.8)	0.180	16.6 ± 2.7 (10.5–21.0)

*Note*: No statistically significant differences were observed between the bilateral measurements (*p* > 0.05), thus the data from both sides were combined. L1 represents the screw trajectory length of the lateral mass screw in the atlas; A1 represents the insertion angulation of the lateral mass screw in the atlas in the outward direction; A2 represents the insertion angulation of the lateral mass screw in the atlas in the caudal direction; L2 represents the screw trajectory length of the pedicle screw in the axis; A3 represents the insertion angulation of the pedicle screw in the axis in the outward direction; A4 represents the insertion angulation of the pedicle screw in the axis in the caudal direction.

The related measurements of the fixation screw trajectories for the atlantoaxial components were as follows: the screw trajectory length of the lateral mass screw in the atlas (L1) was (21.5 ± 2.8) mm, the insertion angulation of the lateral mass screw in the atlas in the outward direction (A1) was (13.2 ± 2.5)°, and the insertion angulation of the lateral mass screw in the atlas in the caudal direction (A2) was (3.5 ± 1.1)°. The screw trajectory length of the pedicle screw in the axis (L2) was (29.8 ± 2.8) mm, the insertion angulation of the pedicle screw in the axis in the outward direction (A3) was (20.7 ± 2.8)°, and the insertion angulation of the pedicle screw in the axis in the caudal direction (A4) was (16.6 ± 2.7)°.

## 4 Discussion

Surgical intervention in the upper cervical spine is inherently challenging due to the limited exposure and the proximity of critical neurovascular structures, including the spinal cord and vertebral arteries. These factors significantly increase the risk of complications and contribute to the technical complexity of such procedures. Consequently, advancements in surgical techniques for the upper cervical spine have been relatively slow, with traditional approaches gradually revealing several limitations. In recent years, numerous researchers have developed various designs for AAOJs [8, 11, 14, 15, 17], with the shared goal of biomimicking the normal morphologic and biomechanical characteristics of the atlantoaxial joint. Despite differences in design, the underlying principles of these AAOJs remain largely consistent. However, most studies have primarily focused on preserving the functional mobility between the atlas and axis, often overlooking critical factors such as the surgical feasibility and ease of the procedure, as well as the ability of the replacement to maintain the overall stability of the occipitocervical junction postoperatively. To address these issues, our research team has undertaken a comprehensive analysis of the anatomical parameters of the atlantoaxial joint over recent years. Based on these repeated measurements, we have made significant modifications to the original design of our AAOJ, resulting in the development of an improved version. This new design not only addresses the functional limitations of previous models but also places a strong emphasis on surgical practicality and the preservation of biomechanical stability after implantation. By considering these critical aspects, our design aims to enhance both the anatomical safety and effectiveness of upper cervical spine surgeries, offering a more robust solution for patients requiring atlanto‐odontoid joint replacement.

### 4.1 Design and Improvement of the Novel AAOJ

The novel AAOJ is fabricated from a medical titanium alloy that exhibits excellent biocompatibility, high in vivo stability, and long‐term wear resistance, aimed at extending its service life. In the design of the new AAOJ, we transitioned from the initial design, which only allowed for rotational movement through a “pivot joint”, to a “ball‐and‐socket joint” that enables not only rotational but also flexion‐extension and lateral bending motions. Additionally, a limiting mechanism was integrated within the ball‐and‐socket joint to ensure that all movements remain within the normal physiological range. Building upon the biomechanical findings of the original AAOJ, we modified the fixation of the atlantoaxial component [18]. The previous design employed a double‐screw fixation on one side of the component, which has been changed to a single large‐diameter screw fixation. This modification simplifies the surgical procedure and reduces the risk associated with multiple screw insertions. Furthermore, the screws used for the fixation of the atlanto‐axial components have been replaced with a newly designed hollow lateral hole screw developed by our research team [19]. During the insertion of these screws, surrounding bone tissue can enter through the side holes and subsequently contribute to new bone formation within the screw. This process increases the contact area between the screw and the atlanto‐axial bone, thereby reducing the risk of screw loosening and enhancing the long‐term stability of the AAOJ after implantation. In addition, we preserved the multi‐porous, hollow design of the base of the axis component from the original AAOJ. This allows bone fragments, which are typically generated during surgery, to be packed into the base through the lower opening. This porous design facilitates bone ingrowth into the base and promotes bony fusion between the base and the axis vertebra [20]. Consequently, this enhances the interface conformity between the component and the bone tissue, while distributing the concentrated stresses on the contact surfaces, ultimately improving the stability of the axis component. However, it is important to note that in a living organism, the contact surface between the prosthetic device and the vertebra is not rigidly fixed but instead exists in a micro‐motion state. Therefore, whether this design can achieve the anticipated theoretical outcomes in vivo remains to be validated through further experimental studies.

### 4.2 Feasibility of Transoral Pharyngeal Approach for the Insertion of Novel AAOJ Implants

The transoral pharyngeal approach is a commonly utilized surgical route for the management of craniovertebral junction and upper cervical spine disorders in clinical practice [21]. However, literature reports indicate that this approach is classified as a Category II incision, with postoperative wound infection rates potentially as high as 50%. Additionally, complications such as cerebrospinal fluid leakage and intracranial infections are possible [22]. Consequently, there exists considerable debate among scholars, both domestically and internationally, regarding the feasibility of performing internal fixation using the transoral route. Studies by Yin et al. [23] and Wang et al. [24] have shown that the postoperative infection rates following the transoral pharyngeal approach are not significantly different from those observed with traditional cervical approaches. Furthermore, recent advancements in surgical techniques have led to an increase in relevant clinical studies. Kandziora et al. [25] and Yin et al. [26] reported successful cases of atlantoaxial fusion using a subarticular atlantoaxial locking plate via the transoral route for the treatment of atlantoaxial instability, with all patients achieving satisfactory clinical outcomes. These studies suggest that, with adequate preoperative preparation, thorough oral antisepsis, minimal intraoperative trauma, and enhanced postoperative oral and pharyngeal care, the postoperative infection rate using the transoral route for internal fixation is unlikely to be significantly elevated. In the present study, we simulated 18 cases of AAOJ replacement surgery and concluded that the transoral route provides a clear surgical field with ample space for necessary surgical maneuvers. Additionally, the novel design of the AAOJ implant has been optimized, enabling the surgical procedure to involve exposure of only the lateral mass of the atlas and the vertebral body of the axis, without the need to expose the lateral vertebral arteries or open the spinal canal to visualize the posterior spinal cord and neural structures. This modification significantly enhances the safety and feasibility of the procedure.

### 4.3 Anatomical Safety of Transoral Pharyngeal Approach for the Insertion of Novel AAOJ Implants

In this study, we evaluated various anatomical parameters during the replacement procedure of a novel AAOJ, both intraoperatively and postoperatively, in order to establish safe window widths and optimal screw insertion points. Specifically, the appropriate width of the anterior arch bone window of the atlas can prevent excessive resection and damage to the lateral masses of the atlas, while the suitable width of the vertebral body bone window of the axis better mimics the dimensions of the base of the odontoid process, thus enhancing stability while ensuring safe placement. The distance between the screw insertion points in the atlas and axis represents the spacing between the center points of the anterior surfaces of the lateral masses of the atlas and the anterior roots of the pedicles of the axis. This parameter can be used to assess the anatomical safety of the screw insertion points.

The atlas is anatomically unique due to its proximity to critical vascular and neural structures. The posterior surface of the atlas is adjacent to the vertebral artery groove and the atlanto‐axial venous plexus. Choosing the appropriate screw length can minimize the risk of damaging posterior structures such as the vertebral artery. The inner surface of the lateral mass forms the vertebral canal wall, housing the spinal cord, while the outer surface forms the wall of the transverse foramen, which contains the vertebral artery. The superior wall is the vertebral artery groove, which houses the vertebral artery, and the inferior wall corresponds to the C1/2 neural root foramen, through which the C2 nerve root passes. Therefore, the screw insertion point, depth, and angle in the atlas lateral mass must be within a safe anatomical range. We utilized a 4.0 mm diameter hollow lateral hole screw for single cortical fixation of the atlas components, with the insertion point located at the center of the anterior surface of the lateral mass. The screw was inserted along the long axis of the lateral mass, that is, perpendicular to the anterior surface of the lateral mass, inclined posteriorly and laterally, thus minimizing the risk of spinal cord, vertebral artery, and cervical nerve injury [27].

The axis, as the second cervical vertebra, presents certain anatomical complexities. The lateral wall of the axis pedicle is in close proximity to the transverse foramen, where the vertebral artery runs. The inferior wall contributes to the formation of the neural root foramen, through which the C3 nerve root passes. The medial wall forms the vertebral canal, which contains the spinal cord. Additionally, due to the suspension of the lateral third of the superior articular facet of the axis, there is a relative reduction in the width of the lower and middle portions of the pedicle, resulting in a relatively weakened anatomical structure in this region [28]. This, in turn, limits the diameter of the pedicle screw that can be inserted into the axis [29]. However, clinical observations have shown that, despite the limited range of screw diameters for axis pedicle screws, excellent grip and reliable biomechanical outcomes are still achievable. Biomechanical studies have confirmed that axis pedicle screws can sufficiently provide the necessary stability for upper cervical spine surgery [30]. Furthermore, there are no significant structures posterior to the posterior surface of the axis pedicle, which makes it feasible to use a 3.5 mm diameter hollow lateral hole screw for double cortical fixation of the axis components. The insertion point is located at the center of the anterior surface of the pedicle, with the screw inserted along the long axis of the pedicle, that is, inclined posteriorly and laterally, passing through the “mechanical core” of the axis pedicle.

Postoperative radiological assessments revealed that all 18 surgeries in this study were successful in terms of prosthesis placement. The internal fixation was well positioned, without any damage to critical vascular or neural structures, and there was adequate bony space surrounding the screws. Therefore, the anatomical and trajectory parameters measured in this study provide safe reference ranges for such procedures. The high success rate of these surgeries further indicates that the transoral pharyngeal approach for the insertion of the novel AAOJ prosthesis is a safe and viable surgical technique. The insertion of screws remains a challenging aspect of the procedure, but with the advancement of spinal navigation technology, the use of navigation‐assisted guidance can further enhance the safety of screw placement during surgery.

### 4.4 Indications for AAOJ Replacement

AAOJ replacement surgery offers a combination of decompression, reconstruction of stability, and preservation of rotational function. Based on the literature, the indications for AAOJ replacement can be summarized as follows: irreducible atlantoaxial dislocations caused by odontoid fracture scarring, rheumatoid arthritis, congenital craniovertebral malformations, and other conditions associated with compression of the medulla oblongata and cervical spinal cord; non‐union of odontoid fractures; and cases requiring odontoid resection and decompression, such as basilar invagination [31]. In cases where the compressive factor originates from posterior structures, posterior decompression and fusion procedures are required [32].

### 4.5 Limitations and Strengths

This study has several limitations that should be acknowledged. Firstly, the sample size is relatively small, with only 18 specimens of the head and neck, which may limit the generalizability of the findings. Secondly, the surgical procedures were simulated on cadaveric specimens without considering certain factors, such as endotracheal intubation, which may influence the surgical approach. Furthermore, the joint mobility, tissue flexibility, and bleeding conditions in cadaveric specimens differ from those in living individuals, all of which could impact the surgical process and outcomes. Thirdly, in order to prevent fractures of the atlas and axis vertebrae, which could result in specimen failure, we opted for a single attempt at screw insertion rather than repeatedly loosening the screws to assess the maximum safe entry angle. This approach may have led to an underestimation of the safe range for the screw trajectory as measured in the radiographic parameters. Lastly, due to the limitations of using cadaveric specimens, we were unable to perform a postoperative range of motion assessment.

The strengths of this study lay in the design of the novel AAOJ, which was based on a solid research foundation, ensuring that its components were more anatomically compatible with the human body. Furthermore, our simulated surgery employed clinically feasible and simplified techniques that effectively reduced surgical complexity. For example, a urinary catheter was introduced through the nasal cavity to retract the soft palate and uvula, thereby improving exposure of the surgical area. Finally, detailed anatomical and imaging parameter measurements provided strong scientific evidence and support for the clinical application of the novel AAOJ.

## 5 Conclusions

Anatomical and radiological findings indicate that the components of the novel AAOJ implant maintain a sufficient safety margin from critical anatomical structures in the human body. Therefore, the transoral pharyngeal approach for the implantation of the novel AAOJ is anatomically safe and feasible.

## Author Contributions

All authors had full access to the data in the study and took responsibility for the integrity of the data and the accuracy of the data analysis. Y.H. designed the study and provided a critical review of the manuscript. S.G.L. and L.Y.J. collected and analyzed the data and wrote the main manuscript. X.J.C. and Z.W.G. provided conceptual advice and statistical analyses and critically revised the paper. J.B.Z., S.G.L., and L.M.Y. performed the surgeries and collected the data. X.J.C. and Z.W.G. prepared figures and tables and revised the initial manuscript. All authors have read and approved the final submitted manuscript.

## Conflicts of Interest

The authors declare no conflicts of interest.
